# Supplementary material for: SVAT: Secure outsourcing of variant annotation and genotype aggregation
Source: BMC Bioinformatics. 2022 Oct 1;23:409. doi: 10.1186/s12859-022-04959-6 (PMC9526274; doi:10.1186/s12859-022-04959-6)
Supplement: Supplementary file 1 — Additional file 1. Supplementary Information for “SVAT: Secure Outsourcing of Variant Annotation and Genotype Aggregation. [file 12859_2022_4959_MOESM1_ESM.pdf]

Supplementary Information for “SVAT: Secure Outsourcing of Variant Annotation and Genotype Aggregation”

Miran Kim[1], Su Wang[2], Xiaoqian Jiang[3], Arif Harmanci[2,*]

1 Department of Mathematics, Hanyang University, Seoul 04763. Republic of Korea.

2 Center for Precision Health, School of Biomedical Informatics, University of Texas Health Science Center, Houston, TX, 77030.

3 Center for Secure Artificial intelligence For hEalthcare (SAFE), School of Biomedical Informatics, University of Texas Health Science Center, Houston, TX, 77030, USA.

*Corresponding Author: Arif Harmanci, arif.o.harmanci@uth.tmc.edu

Full List of VEP Impact Terms used by SVAT

Table 1 shows the full list of VEP impact terms that SVAT uses to annotate SNPs and indels.

Term	CDS	Splice_Region	Splice_Acceptor	Splice_Donor	5p_UTR	3p_UTR	Start_Codon	Stop_Codon	Intron
NOMINAL_IMPACT	0	0	0	0	0	0	0	0	0
intergenic_variant	0	0	0	0	0	0	0	0	0
feature_truncation	0	0	0	0	0	0	0	0	0
regulatory_region_variant	0	0	0	0	0	0	0	0	0
feature_elongation	0	0	0	0	0	0	0	0	0
regulatory_region_amplification	0	0	0	0	0	0	0	0	0
regulatory_region_ablation	0	0	0	0	0	0	0	0	0
TF_binding_site_variant	0	0	0	0	0	0	0	0	0
TFBS_amplification	0	0	0	0	0	0	0	0	0
TFBS_ablation	0	0	0	0	0	0	0	0	0
downstream_gene_variant	0	0	0	0	0	0	0	0	0
upstream_gene_variant	0	0	0	0	0	0	0	0	0
non_coding_transcript_variant	0	0	0	0	0	0	0	0	0
NMD_transcript_variant	0	0	0	0	0	0	0	0	0
intron_variant	0	0	0	0	0	0	0	0	1
non_coding_transcript_exon_variant	0	0	0	0	0	0	0	0	0
3_prime_UTR_variant	0	0	0	0	0	1	0	0	0
5_prime_UTR_variant	0	0	0	0	1	0	0	0	0
mature_miRNA_variant	0	0	0	0	0	0	0	0	0
coding_sequence_variant	1	0	0	0	0	0	0	0	0
synonymous_variant	1	0	0	0	0	0	0	0	0
stop_retained_variant	0	0	0	0	0	0	0	1	0
start_retained_variant	0	0	0	0	0	0	1	0	0
incomplete_terminal_codon_variant	1	0	0	0	0	0	0	0	0
splice_region_variant	0	1	0	0	0	0	0	0	0
protein_altering_variant	0	0	0	0	0	0	0	0	0
missense_variant	1	0	0	0	0	0	0	0	0
inframe_deletion	1	0	0	0	0	0	0	0	0
inframe_insertion	1	0	0	0	0	0	0	0	0
transcript_amplification	0	0	0	0	0	0	0	0	0
start_lost	1	0	0	0	0	0	1	0	0
stop_lost	1	0	0	0	0	0	0	1	0
frameshift_variant	1	0	0	0	0	0	0	0	0
stop_gained	0	0	0	0	0	0	0	1	0
splice_donor_variant	0	0	0	1	0	0	0	0	0
splice_acceptor_variant	0	0	1	0	0	0	0	0	0
transcript_ablation	0	0	0	0	0	0	0	0	0

Supplementary Table 1. The full list of VEP impact terms that are used by SVAT. Each row corresponds to a VEP impact term as identified by the first column. Other columns indicate the impacted element. Each entry on the table with '1' indicates that the term impact the corresponding element on the column and '0' indicates the term does not impact the element.
